# Lameness in Beef Cattle: A Cross-Sectional Descriptive Survey of On-Farm Practices and Approaches

**DOI:** 10.3389/fvets.2021.657299

**Published:** 2021-06-04

**Authors:** Jay Tunstall, Karin Mueller, Dai Grove-White, Joanne W. H. Oultram, Helen Mary Higgins

**Affiliations:** Department of Livestock and One Health, Institute of Infection, Veterinary and Ecological Sciences, University of Liverpool, Neston, United Kingdom

**Keywords:** lameness, beef, locomotion, cattle, perceptions, welfare, prevalence, farmer training

## Abstract

Cattle lameness is a concern to the United Kingdom (UK) cattle industry, negatively impacting upon welfare and production. Previous work involving one small study (*n* = 21) has identified that some UK beef farmers underestimate lameness prevalence, but also that farmers vary in their perception of the impact of lameness. Knowledge and skills of farmers were identified as a potential concern, and farmer-reported barriers were identified. However, the extent to which these views can be extrapolated is unknown. Therefore, the aim of this study was to produce descriptive results of UK beef farmer lameness-related activities concerning lameness identification, examination, treatment, and prevention. Questionnaires were circulated online and via post. Postal questionnaires were sent to registered Approved Finishing Units (a specific cohort of beef fattening units subject to strict biosecurity measures as part of UK bovine tuberculosis control) and a stratified sample of all registered beef enterprises in England and Wales. Online questionnaires were circulated on social media and via targeted emails asking selected industry bodies and veterinary practices to distribute to farmers. Descriptive results were produced, and thematic analysis was performed on free text responses. There were 532 usable responses, with most farmers self-reporting their current lameness prevalence as zero (mean 1.2%, range 0–20%). Most respondents did not locomotion score cattle, and most reported that it was not safe to examine feet. Most farmers did not use a foot bath, but of those who did, formaldehyde was the most commonly used product. Some farmers reported use of antibiotic foot baths. Most farmers reported dealing with lame animals within 48 h, but some only dealt with severe cases, and some felt that lame animals would get better by themselves. To deal with animals that have an ongoing lameness problem, transportation to slaughter was considered an option by 35% of farmers. It is worth noting, however, that the majority of lame animals would be precluded from transport under UK legislation. Farmers reported staff shortages, as well as a lack of time, training, and knowledge as barriers to lameness prevention and control. Overall, these results suggest that farmers may be underestimating lameness. Diagnosis is likely to be challenging, with unsafe facilities for lifting feet. The reported high threshold by some farmers for attending to a lame animal is a cause for concern, negatively impacting upon animal welfare, but this is also likely to have negative consequences for animal performance and farm profitability. Many participants in this study expressed a desire for farmer training in several aspects relating to lameness prevention and control, and this represents an opportunity for further knowledge exchange regarding lameness in beef cattle.

## Introduction

Cattle lameness is a concern to both the United Kingdom (UK) dairy and beef sectors, due to its impact on welfare and on production (1, 2). Its economic impact is well recognized in the dairy sector (3). Despite this, the mean farm level prevalence of lameness in UK dairy cattle has remained over 20% for over 20 years (4–7), with a recent estimate suggesting a mean farm level prevalence of 32% (4). Although it is acknowledged to be one of the most important disease processes in beef production (8), there is less known about its impact or prevalence in this sector. Canadian work suggested notable financial losses due to lameness in feedlot systems (8). Unpublished work by the authors suggests an estimate of the UK mean farm level prevalence of lameness to be 8.3% (95% CI 5.58–10.99) for finishing cattle and 14.2% (95% CI 7.83–20.63) for suckler cows.

There are a number of dairy sector studies identifying risk factors for lameness, some of which may be within a farmer's control. Herd size (9), duration of housing or grazing access (5, 10–14), depth of bedding material (5, 9, 15, 16), provision of deep litter yards (5, 13, 17), stocking density (18), footbath provision, routine foot trimming provision and concentrate feeding (4) all have been associated with lameness risk for dairy cattle. However, in terms of beef cattle, there is a relative paucity of research in this area.

Many risk factors for lameness are under the control of farmers, and it is essential to understand farmers' perceptions and their current role in the treatment and prevention of lameness because this can have a major influence on animal health and welfare. For example, it has been suggested that dairy farmers might underestimate lameness on their farms (19, 20), and beef farmers may do the same (21), which may affect the importance that they place on lameness prevention, and may inhibit treatment if lame animals are not identified.

A recent qualitative and quantitative study that included in-depth interviews with 21 UK beef farmers investigated their perception of the impact of lameness on production, animal welfare, and on farm staff themselves (21). In this study, some farmers reported leaving lame animals untreated as long as it does not become “too bad,” or as long as a cow still conceives. Lesion identification and foot trimming ability, appropriate use of medicines, prompt detection of lameness, and decision making relating to culling a lame animal have all been identified as issues for some beef farmers (21). Furthermore, some farmers stated a lack of time or lack of capable staff, or felt that they could not justify investing in their facilities in order to better treat or prevent lameness, due to expecting a poor return on investment (21). In addition, this same study also involved a researcher locomotion scoring the cattle and comparing their estimate of lameness prevalence to the beef farmer self-reported estimate, that same day. The findings showed that 19/21 beef farmers underestimated lameness prevalence on their farms compared to the researcher. These issues all have the potential to compromise welfare within the UK beef industry, and also decrease the efficiency of the industry, although as previously discussed, the economic and production impact of lameness in beef cattle is yet to be robustly established.

However, as the published literature described in the previous paragraph only covers a small number of farmers and is also, to our knowledge, the only UK study on perceptions of lameness in beef cattle, more research was required to build on these findings and establish to what extent the results from this research were applicable to the wider UK beef farmer population. The intent of this study was therefore to use the findings from previous research (21) to inform the design of a cross-sectional questionnaire that could be deployed on a considerably wider scale, thereby yielding quantitative descriptive data that would provide further insight into the population at large. The specific aims of this study were to produce descriptive results of UK beef farmer lameness-related activities, enabling (i) insights into beef farmer perceptions of lameness prevalence on their farms; (ii) exploration of lameness identification, examination, and treatment choices on UK beef farms; (iii) identification of barriers to lameness treatment and prevention, and (iv) investigation of beef farmer training and confidence regarding lameness identification, treatment, prevention, and control.

## Methods

This study was approved by the University of Liverpool Research Ethics Committee (VREC 533).

### Questionnaire Design

The questionnaire was based on the findings from the qualitative study involving beef farmer interviews (21) and informed by the literature and clinical experience of the research team. The question topics were initially proposed by JT, and discussed with all members of the research team at length (DGW, HMH, KM, and JO). This enabled draft questions to be constructed by JT, which were scrutinized and further refined in discussion with the research team, leading to the creation of the near final version. The questionnaire was grouped into sections that covered demographic information, ability, and method of management and treatment of lameness, dealing with chronically lame animals, training of farmers, lameness prevalence, and perceived barriers to lameness control. There were 32 questions, of which 20 were closed and 12 were free text replies.

The near-final version of the questionnaire was then initially piloted in its paper format with a convenience sample of 10 beef farmers. Minor alterations were made before the online version was created, which was then successfully piloted with two beef farmers. The pilot surveys enabled an estimate of the time taken to complete the questionnaire to be established, which was ~10 min. The responses from the pilot study were not included in the analysis.

The questionnaire was designed to require mainly tick box or Likert scale (22) responses, with some open questions to provide product names, or occasionally reasons for decisions. Free text boxes were provided for some questions to enable those wishing to expand on their answers to do so.

The online questionnaire provided automated skipping of unrelated questions, for example, farmers who stated they did not treat lame animals were not asked about products they used. For the online version, one question deemed to be essential (the number of animals present on the farm) was enforced, but all other questions could be skipped. Multiple choice answers or topics within questions were randomized (where the responses were not logically ordered, such as age category). This randomization was not performed with the paper version. However, guidance regarding skipping unrelated questions was provided in the text.

Both online and paper versions provided a participant information sheet and requested consent, which was mandatory for online respondents to continue. The paper questionnaire provided instructions for a change of mind on individual questions once marked, whereas the online version allowed respondents to change their selection by selecting an alternative option. The postal questionnaire is available in the Supplementary Material. Respondents were offered the opportunity to win a pair of wellington boots as an incentive to complete the questionnaire. Those wishing to enter were asked to provide their contact details, which were used solely for the purposes relating to the prize.

### Identification and Recruitment of Farmers

The target population was defined as UK beef farmers, working with either breeding beef heifers or cows, or weaned cattle being reared for beef production at any stage in the process (e.g., including stores, fattening or finishing cattle). Farmers that owned, managed, or worked on a farm were considered eligible, regardless of herd size or cattle numbers. Farmers involved with more than one eligible farm were asked to answer questions for the unit that they had the most “hands on” involvement with the cattle.

#### Online Circulation

The questionnaire was uploaded to Qualtrics online survey platform (Qualtrics, Provo, Utah, USA) in February 2019 and remained open for 12 months. A link was added to the University of Liverpool social media outlets that was shareable by other social media users. The link was also emailed to targeted industry bodies and veterinary practices (*n* = 10 and *n* = 50, respectively, identified via press, online media, and personal contacts) with the request to circulate to relevant farmers. The link provided a participant information page and access to the online questionnaire, as well as details of how to request a paper copy of the questionnaire.

#### Postal Circulation

Paper questionnaires were distributed via post. The first launch was to all (*n* = 340) farmer addresses listed as Approved Finishing Units (AFUs) in England and Wales, obtained via the UK Government Bovine TB website (23), and occurred in April 2019. These farmers received a postal reminder in May 2019.

The Animal and Plant Health Agency (APHA) provided access to a list of registered beef holdings in England and Wales (*n* = 46,999). Access to data for Scotland and Northern Ireland was not granted. A random sample of 2,000 holdings, stratified by farm type (suckler cow or non suckler cow), county registered in, and herd size (1–9, 10–29, 30–49, 50–99, 100–149, 150–499, and 500+ cattle), was selected using STATA/MP 14.1 (Statacorp, College Station, Texas, USA) for Windows. Selection took account of the relative proportions of farm numbers in a county and herds within a certain size category (for example, if a county contributed 3.5% of farms to the total, the target number to be selected from that county would be 3.5%). Paper copies of the questionnaire were sent to the selected 2,000 farmers in December 2019. The paper questionnaire also provided a link to the online version, offering recipients the choice to complete it using their preferred method. No postal reminders were sent to this cohort.

All postal questionnaire recipients received a postage paid, self-addressed envelope, along with a pen to encourage completion and return.

Due to the multiple channels of questionnaire distribution, the snowball nature of online social media circulation, and the ability of postal questionnaire recipients to complete online using the link provided, it was not possible to accurately determine a response rate for the questionnaire because the denominator (number of eligible people who received the questionnaire) was unknown.

### Data Analysis

Once the questionnaire was closed, data from both Qualtrics and the paper questionnaire responses were uploaded to Microsoft Office Excel (Microsoft 2016). The data were then uploaded to STATA/MP 16.1 (Statacorp, College Station, Texas, USA) for Windows for descriptive and statistical analysis. An exploratory analysis, in terms of plausible associations between variables, was investigated using Fisher's exact test, with α = 0.05. To adjust for multiple hypothesis being tested (which was 20), a Bonferroni correction was applied resulting in α = 0.0025. Data were exported to Minitab statistical software (Minitab 18, PA, USA) for the purposes of graphical representation. Thematic analysis was performed on free text replies, i.e., the qualitative data as described by Braun and Clark (24) using NVivo Pro qualitative data analysis software (QSR international Pty Ltd. Version 12, 2018). Thus, using the software, the text in the data was coded and then merged into themes, which together encapsulated the key messages contained in the free text replies. Exemplar quotations are used to illustrate the constructs identified. When there was a choice of more than one quote, the authors made a judgment as to the most insightful quote to present in the Results Section.

## Results

### Responses

There were 398 postal responses, six of which were returned blank and seven declared that they did not have any cattle of interest (breeding cows or heifers, or weaned cattle that are being reared for beef production), and as such were ineligible. There were 200 online responses, 50 of which did not get beyond giving consent, and 1 response was ineligible due to not having any cattle of interest. As this study was aimed at UK farmers, one response each from France and Ireland were considered ineligible. Ineligible responses were removed, leaving 385 postal responses and 147 online responses. Postal and online responses were then combined to provide 532 total eligible responses.

Partial completion was possible for both postal and online questionnaires. The only compulsory online questions were an initial consent box and a declaration of the number of animals of interest (serving to confirm eligibility as well as demographic information). If part of a response was not possible to decipher (illegible etc.), that part was removed, but the remaining response was retained. The number of responses varied across different questions. The number of respondents answering each question is indicated in brackets throughout this manuscript. Exact wording of questions has been shortened for illustrative purposes on some figures; however, the question (Q) numbers have been provided in figure legends where appropriate for clarity, and refer to the question numbers in the original questionnaire (provided in full in the Supplementary Material).

### Respondents

**Figure 1:**
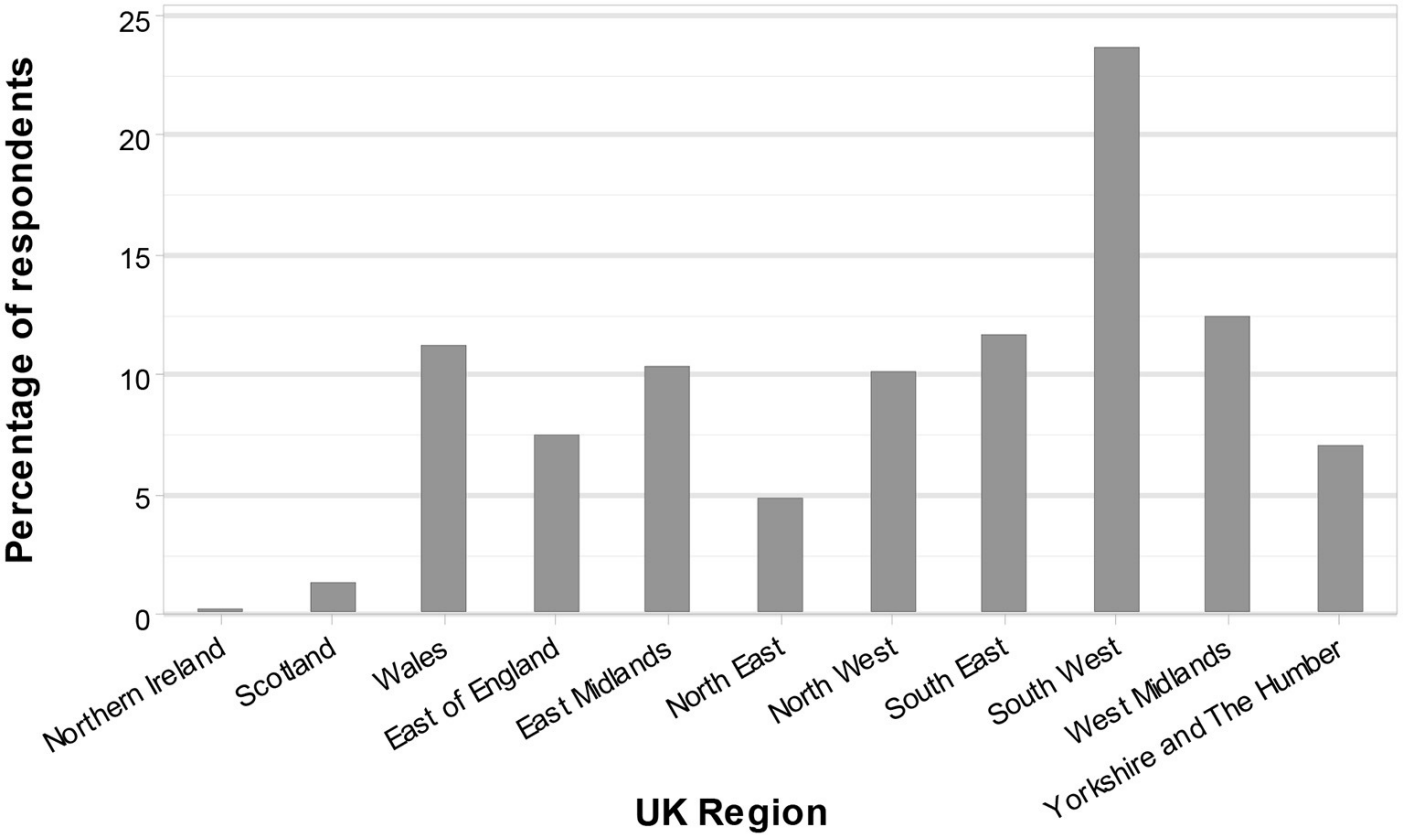
Farm location of respondents, by region, as a percentage of respondents (Q29, *n* = 483). Regions utilized are Northern Ireland, Scotland, Wales, and the nine official regions of England. There were no respondents from Greater London.

**Figure 2:**
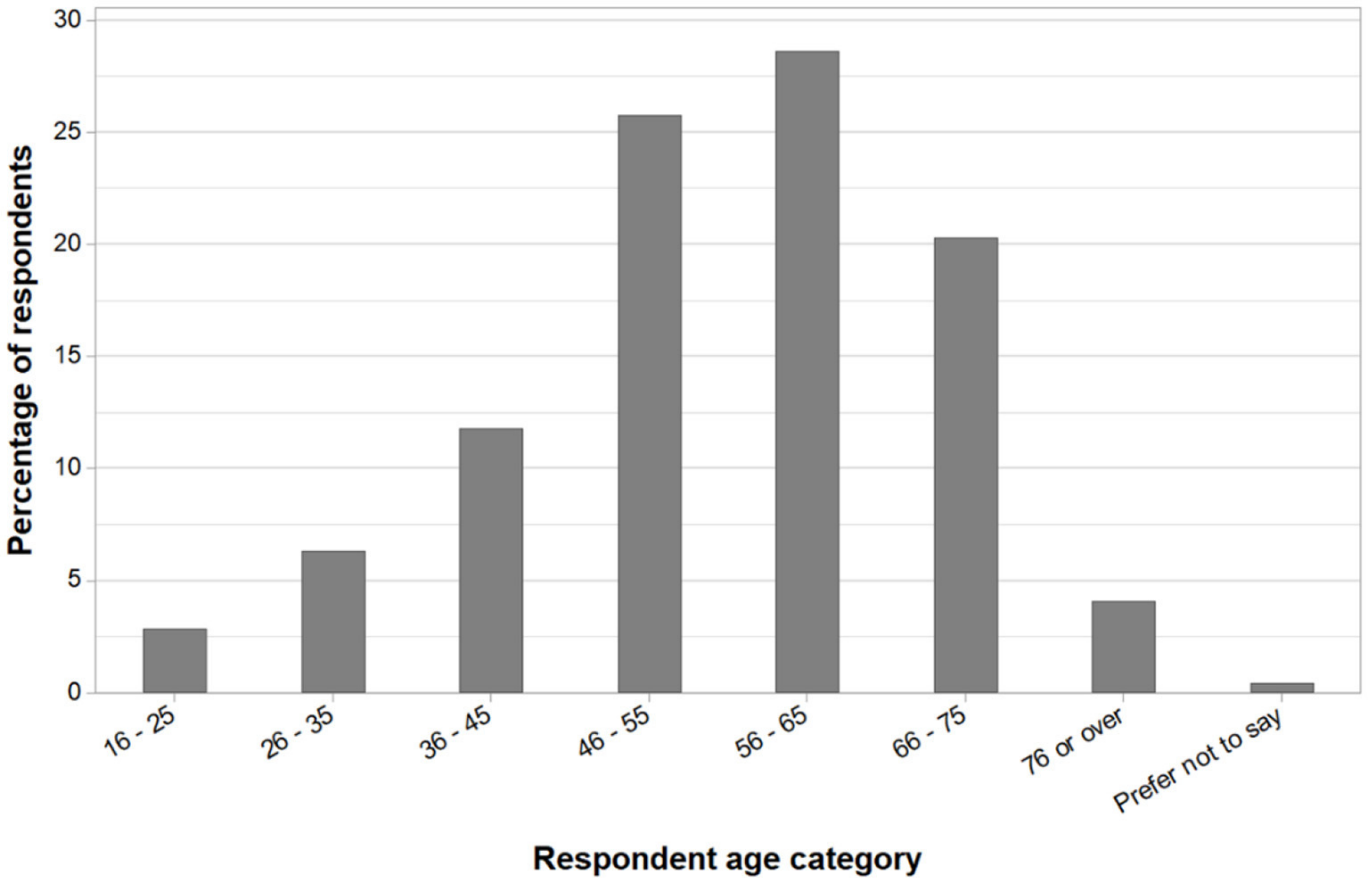
Age of respondent was grouped into categories, as a percentage of respondents (Q27, *n* = 493). There were no respondents in the category “15 years or less.”

In total, 483 respondents answered the question pertaining to their location and 87% (422/483) of these were farming in England, 11% (54/483) in Wales, 1% (6/483) in Scotland, and 0.2% (1/483) in Northern Ireland (Figure 1). The gender split of responses was 82% (406/494) male and 18% (88/494) female. The age range of respondents (*n* = 493) is shown in Figure 2, with a distribution of participants around a modal age of 56–65 years apparent. No respondents selected the 15 years or less category.

Of 485 responses, 83% (402/485) described themselves as the farm owner, 17% (81/485) as a farm manager, and 17% (82/485) as a farm worker. Moreover, 19% (90/485) selected “other” and entered a free text reply, either exclusively (55/90), or in addition to other options (35/90). “Other” free text replies included tenant farmer, managing partner, herdsperson, small holder, retired farmer, farmer's son/daughter, and cattle owner. Respondents could select multiple answers.

Participants were asked to select a response relating to their main source of income, to which 60% (290/481) declared that beef farming was either their main source of income, or an equal top share with another source. Another 10% (47/481) stated that their main source of income was derived from livestock, but not beef farming, and 6% (27/481) stated that arable farming was their main source of income. Neither livestock nor agriculture was the main source of income for 24% (117/481) of respondents.

Farmers were asked, regarding the beef cattle component of the farm in question, whether they were responsible for long-term farm planning, day-to-day management decision making, or day-to-day stockmanship/animal care, and were requested to select all options that apply. Of 487 responses, 86% (420/487), 87% (425/487), and 88% (428/487), respectively, declared responsibility for these three areas.

When considering some specific management systems, the vast majority [93% (457/493)] were not organic, 7% (34/493) declared their cattle to be classified as organic, with 0.4% (2/493) of respondents unsure. About two-thirds [62% (308/496)] did not consider rearing and selling beef breeding stock to be a major part of their business. There were 37% (182/496) who did, and 1% (6/496) of farmers were unsure if it was a major part of their business.

### Number of Animals and Lameness Prevalence

**Figure 3:**
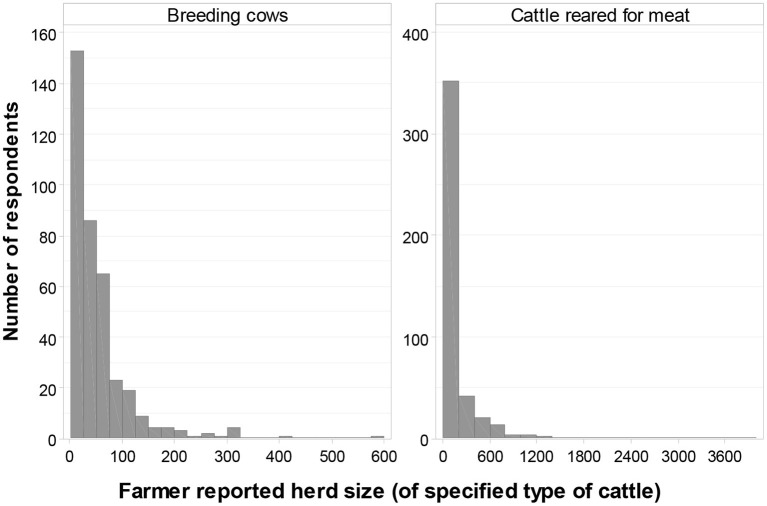
Farmer-reported number of beef cattle of interest on their farm, subdivided by type of cattle: (i) breeding cows and pregnant heifers and (ii) cattle reared for meat, from weaning to slaughter (Q16, *n* = 376 and *n* = 441).

**Figure 4:**
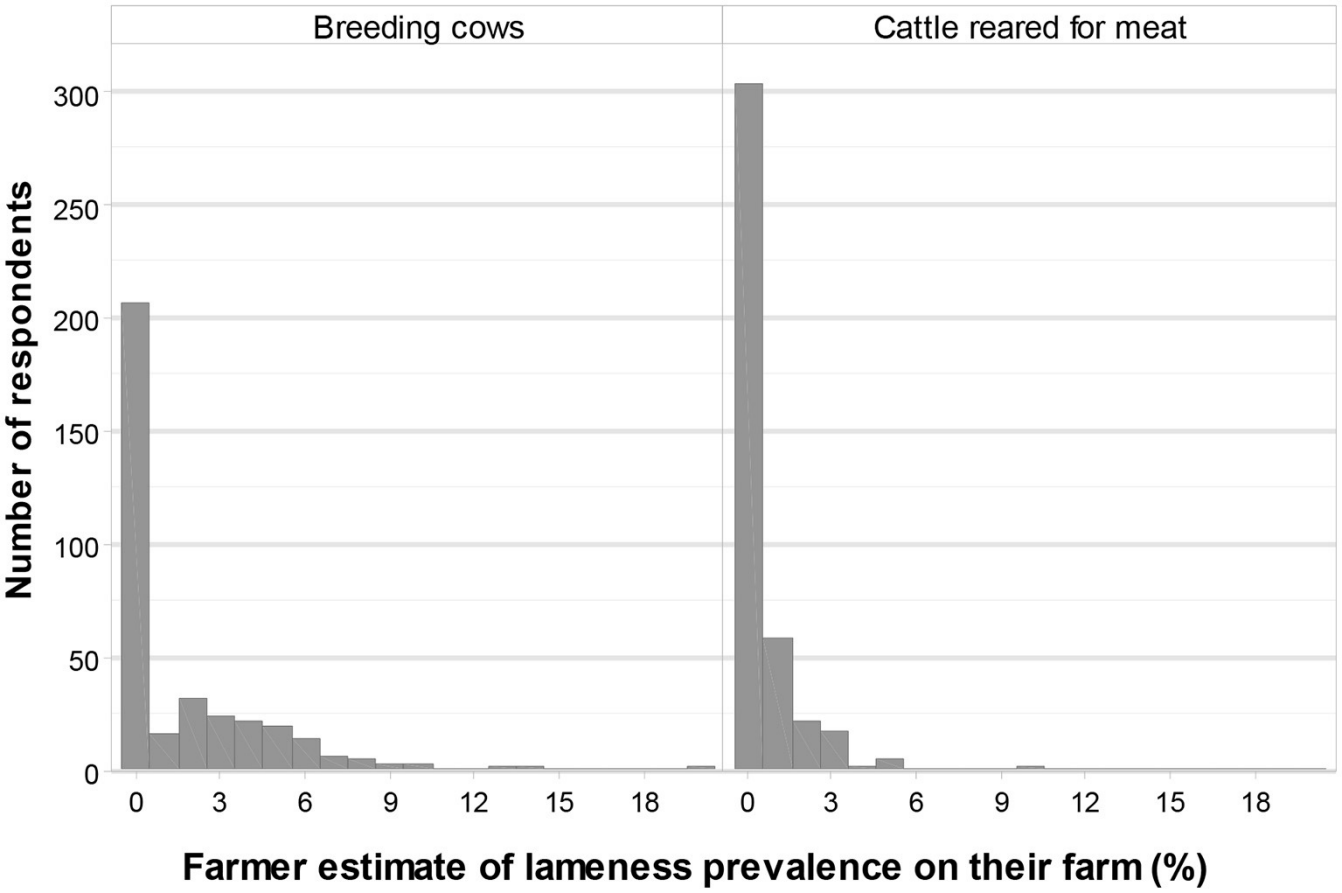
Farmer-reported lameness prevalence on their own farm, subdivided by type of cattle: (i) breeding cows and pregnant heifers and (ii) cattle reared for meat, from weaning to slaughter (Q16, *n* = 359 and *n* = 413).

A total of 71% (376/532) of respondents indicated that they had beef breeding cows/heifers on their farms. Furthermore, 95% (359/376) of these farmers stated the number of animals that they believed to be lame on their farm, currently. The mean number of breeding cows (which also included any pregnant heifers) was 50 (range 1 to 600), with a total of 18,653 animals (Figure 3). Most farmers reported having no lame animals (median 0%, mean 2%, range 0–20%, Figure 4).

A total of 83% (441/532) of farmers indicated that they reared cattle on their farms for meat, from weaning to slaughter. Furthermore, 94% (413/441) of these farmers stated the number of animals that they believed to be lame on their farm, currently. The mean number of animals reared for meat, from weaning up to slaughter, was 155 (range 1–4,000), with a total of 68,333 animals (Figure 3). Most farmers reported having no lame animals (median 0%, mean 0.6%, range 0–17%, Figure 4).

### Lameness Identification, Examination, and Treatment

#### Locomotion Scoring and Lifting Feet for Examination

Of respondents, 89% (422/475) declared that they do not perform any locomotion scoring themselves, and 3% (15/475) specified that they were unsure if they locomotion score. Of the just 8% (38/475) that stated that they do locomotion score, scrutiny of the free text answers associated with this question revealed that, for the majority, this meant being alert to lameness during their daily interaction with the cattle. The answers of only six respondents suggested that any structured locomotion scoring may take place (and then once or twice a year).

Of respondents, 35% (177/513) stated that they always treat lame beef cattle themselves, and approximately half (52%, 266/513) stated that they sometimes do, with 14% (70/513) never treating lame beef cattle themselves. When farmers were asked about lifting the front and back feet of cattle, most farmers selected that lifting and examination was possible, but was generally not safe for either the animal or the person. This percentage of farmers was 56% (286/510) for front feet and 57% (290/511) for hind feet. Lifting and examination was considered possible and generally safe by 13% (68/510) and 11% (58/511) of farmers for front and back feet, respectively. Lifting and examination was considered not possible by 31% (156/510) and 32% (163/511) of farmers for front and back feet, respectively.

#### Treatments

**Figure 5:**
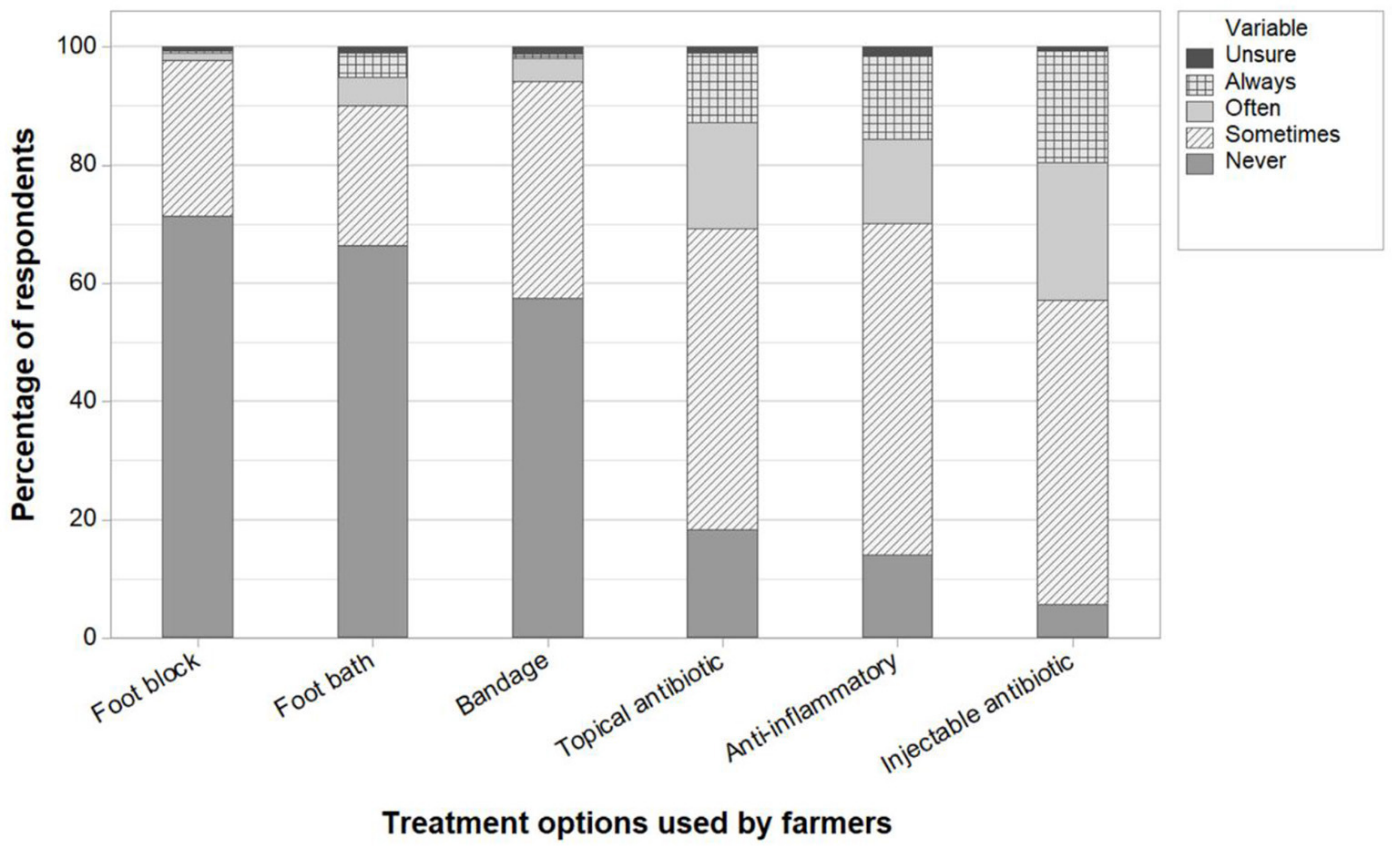
Farmer responses to frequency of use of potential treatment options, as a percentage of respondents (Q5 and Q6, *n* = 404, 414, 406, 407, 428, and 434, respectively).

**Figure 6:**
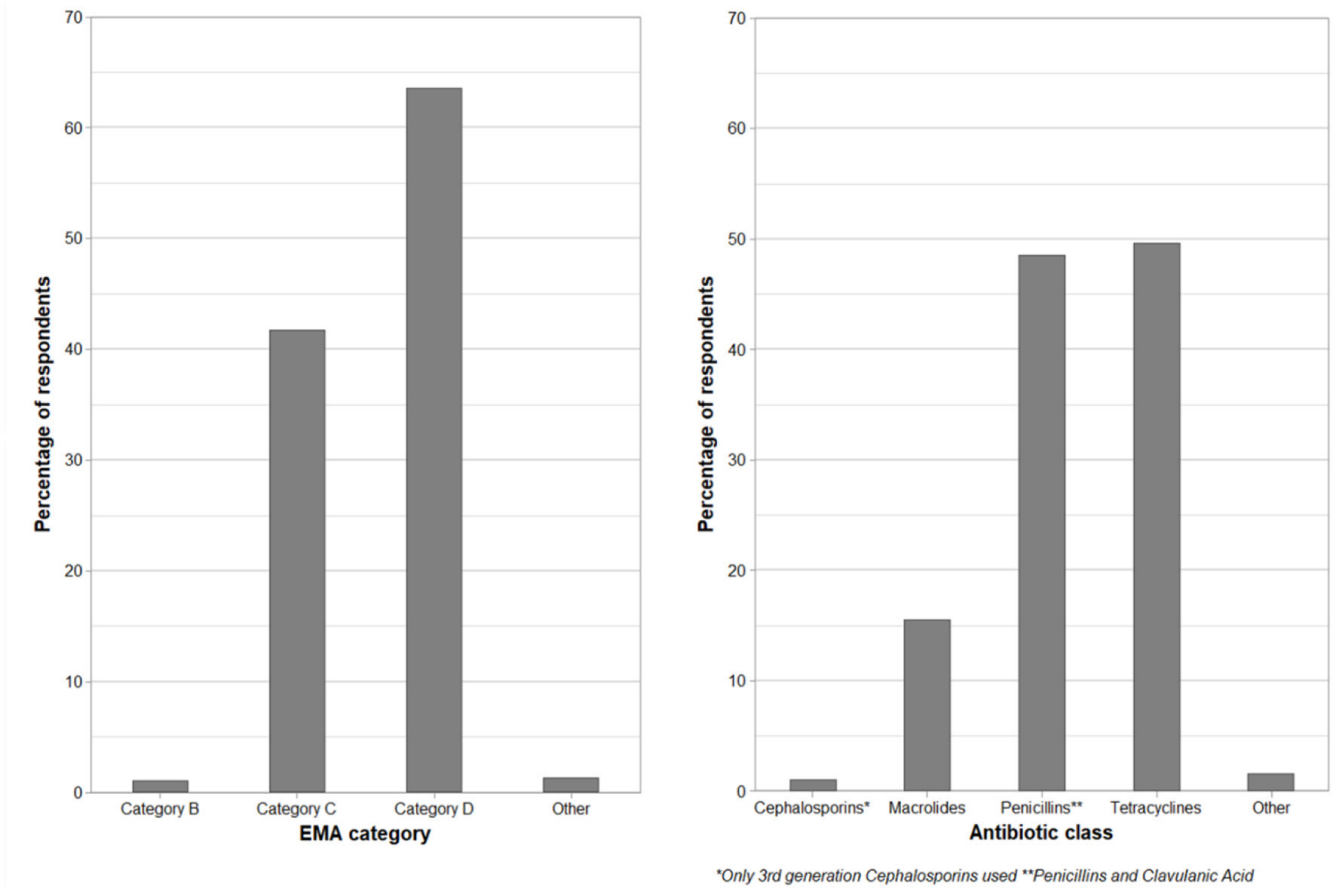
Left-hand panel: Farmer self-reported injectable antibiotics used to treat lame beef cattle, as a percentage of respondents, grouped by the European Medicines Agency categorization (25). Right-hand panel: Same data but grouped by antibiotic classification. “Other” includes five unspecified antibiotics, and one farmer used florfenicol. Farmers could state multiple antibiotics. There were 3% (11) of farmers that reported that they do not use an injectable antibiotic (Q8, *n* = 381).

Farmers who treated lame animals themselves were asked about treatments they use for lame beef cattle (with an emphasis on their own regimes, rather than what an external professional might use on their farm). Responses were given on a five-point Likert scale of “never” to “always,” including an option to state if they were unsure. Responses are shown in Figure 5. It is worth noting that in the UK, farmers can legally administer antibiotics and other “prescription only” medicines to lame cows so long as they are “under the care of a veterinary surgeon” but without the need to contact their veterinary surgeon before treating every individual case. Over half of farmers declared that they never used foot blocks (71%, 288/404), foot baths (66%, 275/414), or bandages (57%, 233/406) on their farms. There were 407, 434, and 428 responses regarding topical antibiotics, injectable antibiotics, and anti-inflammatory products, respectively, with these products being used at least sometimes by the majority (>80%) of respondents. These farmers were also asked to name the two most common antibiotic injection products they use to treat lame beef cattle (Figure 6).

The most common antibiotic class was tetracycline, with 50% (189/381) of respondents stating that they use a product from this class, followed by 49% (185/381) stating that they use a product from the penicillin and clavulanic acid class. There were 15% (59/381) of respondents who stated they use macrolides, and 1% (4/381) of farmers using 3rd- or 4th-generation cephalosporins. An amphenicol was used by 0.3% (1/381) of farmers, and an unnamed antibiotic was used by 1% (5/381) of farmers. Non-steroidal anti-inflammatory drugs (NSAIDs) were listed by 14% (53/381) of respondents, despite the question asking for injectable antibiotic use. Some farmers only listed one antibiotic (or multiple from the same class), and some listed more than two. A minority of farmers (3%, 11/381) specified that they do not use any antibiotics of their own accord.

**Figure 7:**
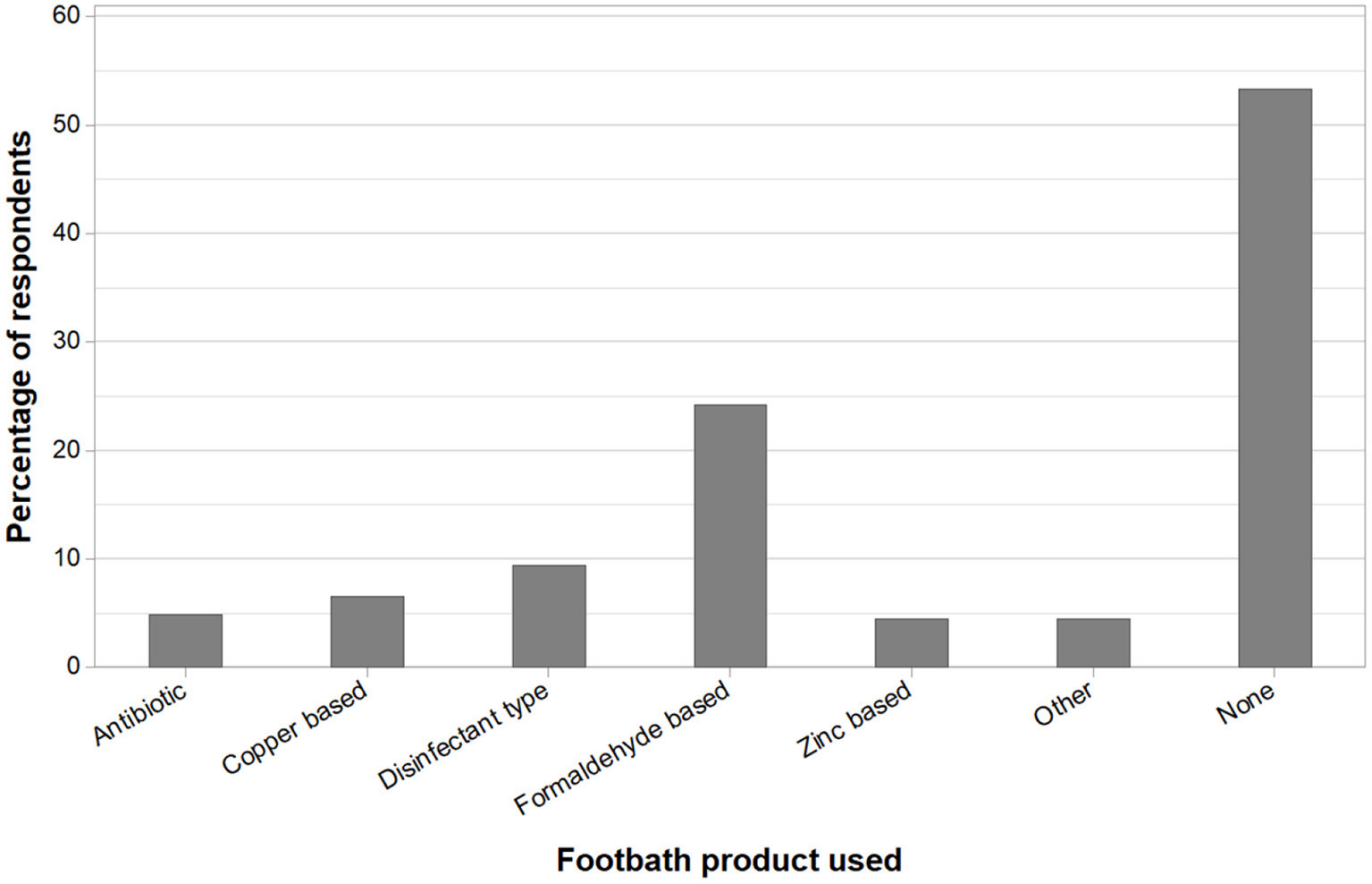
Farmer self-reported foot bath products, as a percentage of respondents. Farmers could state multiple products. “Other” included: a water footbath, products that could not be remembered, or farmer stating that the product used would be based on advice at the time of foot bathing. There were 53% (130) farmers that reported they do not use a footbath product (Q9, *n* = 244).

Respondents were also asked to provide names of any footbath products they use, with multiple answers allowed. Figure 7 indicates the footbath products used, with the most popular product being formaldehyde, used by 24% (59/244) of farmers who responded to this question, followed by 7% (16/244) and 5% (11/244) of farmers using copper-based and zinc-based products, respectively. Of the respondents, 9% (23/244) used another disinfectant-based product (including household disinfectant products, chlorhexidine, an iodophor-based disinfectant, salt water, or an unnamed disinfectant) and 5% (12/244) used an antibiotic foot bath, all of whom reported using a lincosamide product (which are not licensed for this use). The 5% (11/244) of farmers in the category “other” included those using a water foot bath, a product that could not be remembered, or a product that would be selected dependent on advice at the time. A further 53% (130/244) of farmers stated that they did not use a footbath product. Some farmers provided more than one answer to this question.

#### Promptness of Examination of Lame Animals

**Figure 8:**
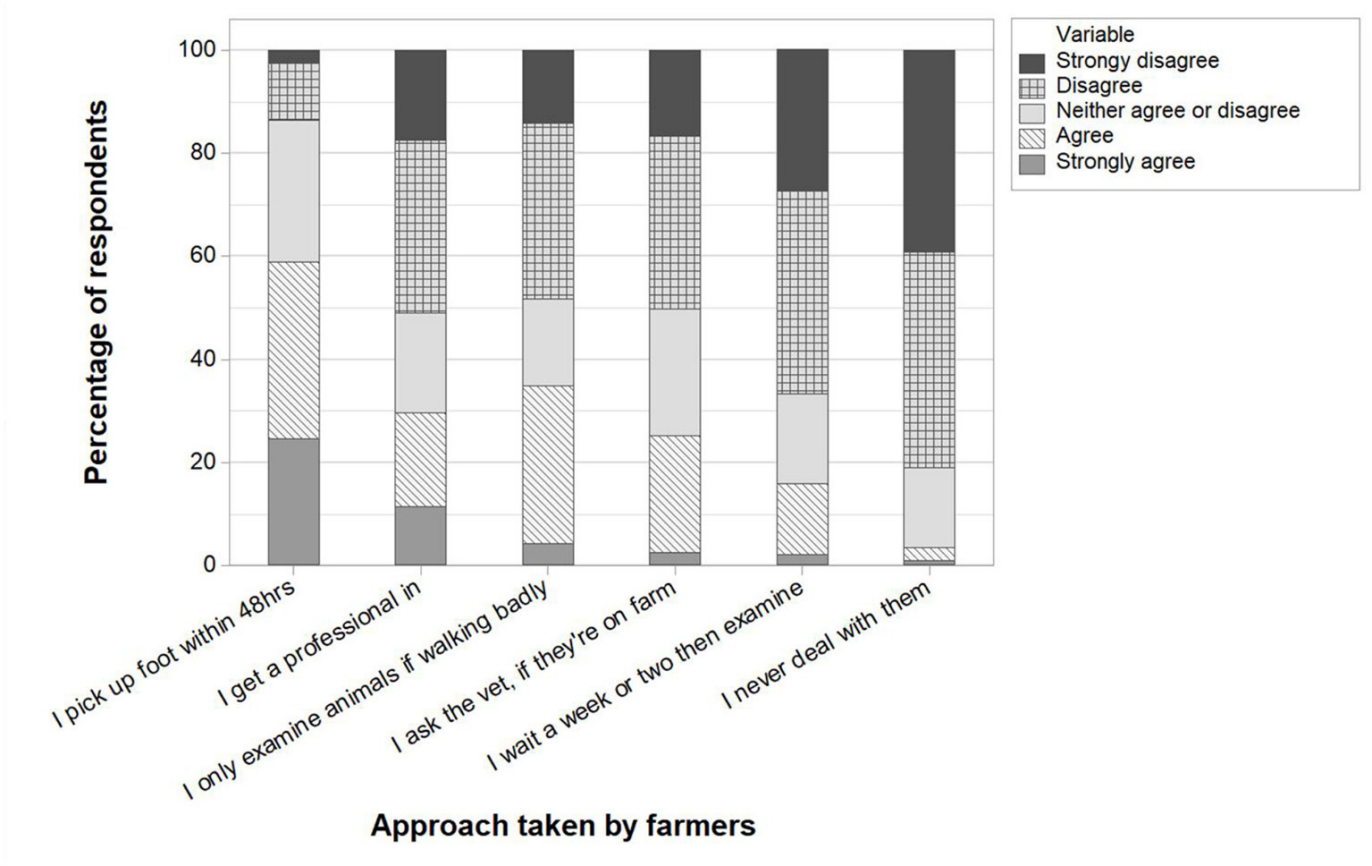
Farmer responses to the level of agreement with approaches that might be taken to deal with lame animals, as a percentage of respondents. Exact wording in the questionnaire was as follows (from left to right on the graph): I pick up the foot of a lame animal within 48 h (*n* = 483); I personally never pick up feet, but I get my vet or foot trimmer to do it as soon as possible (*n* = 478); I only examine animals if they are walking quite badly (*n* = 477); I ask the vet to look at a lame animal, but only if the vet happens to be on farm already (*n* = 473); I give lame animals a week or two before examining them, to see how they do (*n* = 476); I never deal with lame animals, as they get better by themselves (*n* = 473). (Q18).

Respondents were provided with six statements regarding dealing with lame animals in general and asked to declare a level of agreement, from strongly agree to strongly disagree (Figure 8). While the majority of respondents (59%, 284/483) either agreed or strongly agreed that they would “pick up the foot of a lame animal within 48 h,” a minority (3%, 16/473) either agreed or strongly agreed that they “never deal with lame animals, as they get better by themselves.”

**Figure 9:**
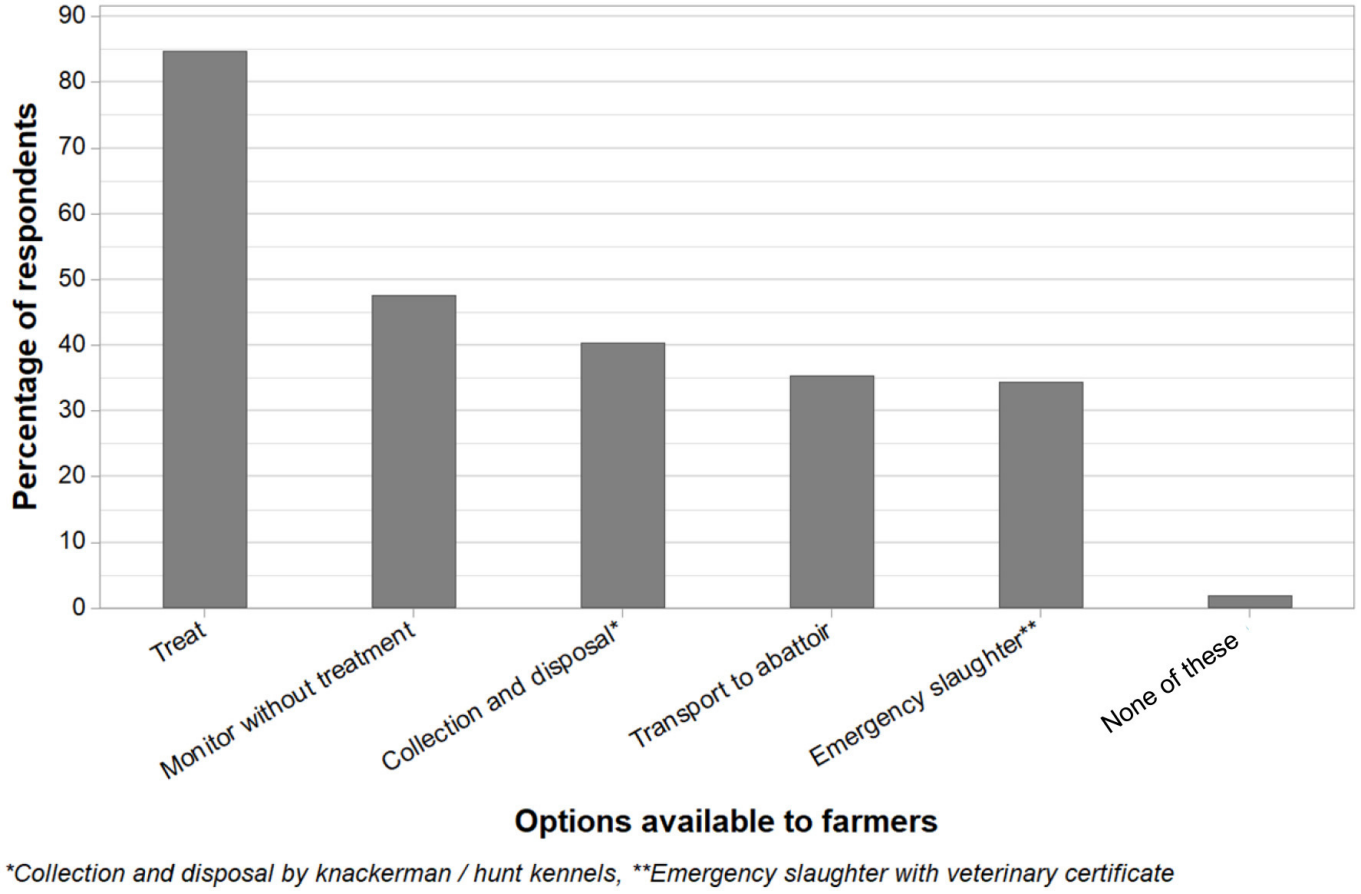
Farmer self-reported options available to them to deal with animals that have ongoing lameness, as a percentage of respondents. Farmers could select multiple answers [except if they select “None” (Q10, *n* = 501)].

Farmers were also asked to select answers that they feel are available to beef farmers to deal with animals that have *ongoing* lameness (i.e., chronic cases), and to select as many as they feel apply (Figure 9). Of respondents, 85% (424/501) felt that they might arrange treatment and keep the animal on the farm; however, 48% (238/501) felt that they might monitor the animal and allow time to recover, without treatment. Calling a knackerman or hunt kennel for collection and disposal was considered an option by 40% (202/501) of farmers, and transporting the animal to a slaughterhouse was considered an option by 35% (177/501) of farmers. Calling the vet for an emergency slaughter certificate (on farm slaughter) was considered an option by 34% (172/501) of farmers, whereas 2% (9/501) of respondents felt that none of these options were available to deal with beef animals with ongoing lameness. Of these nine farmers, two left comments suggesting that they would get a professional in, and three suggested that they do not experience ongoing lameness problems in their cattle. Four provided no further comment.

### Barriers to Treating lameness

Farmers were asked about what, if anything, prevents them from treating lameness or makes treatment difficult, and were provided a free text box to respond, to which 396 farmers responded. Four major themes were identified: (A) facilities and location; (B) staff, time, and knowledge; (C) concerns over drug use; and (D) no barriers to treatment.

Theme A, facilities and location, was the most frequently mentioned theme, with four sub-themes, namely, (i) location of cattle, (ii) inadequate facilities, (iii) dangerous animals, and (iv) safety of staff. One farmer highlighted that:

“*An old crush is up at* (site away from main farm)*, but treatment is not easy*.”

Another farmer highlighted that:

“*Sometimes animals are too wild*.”

Theme B (staff, time and knowledge) had four sub-themes, namely, (i) staff availability and time, (ii) staff ability, (iii) knowledge on lameness, and (iv) perceived requirement to treat lameness. A number of farmers mentioned that staff availability in general was a problem, whereas others mentioned the ability of their staff or themselves, with either age, ill health, or knowledge being a concern, with one stating:

“*More training on lameness in cattle is needed*.”

Some farmers stated that they simply did not have any to treat, whereas another stated:

“*They get better by themselves 90% of the time*.”

Theme C, concern over drug use, was mentioned generally in terms of withdrawal period concerns:

“*Drug withdrawals make decisions hard close to slaughter*.”

However, two farmers did consider responsible use of antimicrobials:

“*I don't want to overuse antibiotics*.”

Lastly, in terms of theme D, 20% of respondents stated that nothing prevents treatment of lame beef cattle, or makes it difficult. Some also highlighted the welfare importance:

“*Nothing prevents me, it has to be treated quickly for the welfare of the animal*.”

### Barriers to Preventing Lameness

In addition to the previous section, which asked farmers what may hinder them *treating* lame animals, farmers were also asked about what, if anything, stops them from *preventing* lameness in beef cattle, or makes prevention difficult, and they were provided with a free text box in order to reply. This question received 319 responses, and three themes were identified: (A) facilities and location; (B) staff, time, and knowledge; and (C) no barriers to prevention.

Theme A, facilities and location, again had four sub-themes: (i) location, terrain, and weather; (ii) inadequate facilities; (iii) dangerous animals; and (iv) safety of staff. A number of farmers mentioned weather, particularly wet weather, muddy gateways, and trough areas and the presence of stones, and some mentioned wet housing conditions as an issue. Several also mentioned issues with either unsuitable facilities, such as handling facilities, or facilities likely being too far from the cattle. One highlighted that they felt footbaths could not be used:

“(You) *could footbath in a dairy situation, but not our beef unit*.”

Theme B (staff, time, and knowledge) had four sub-themes, namely, (i) staff availability and time, (ii) staff ability, (iii) knowledge of lameness topics, and (iv) perceived requirement to prevent lameness. Some farmers mentioned staff shortages in the livestock sector, and some highlighted time as an issue, including one farmer who stated:

“*Time. In an ideal world we would do more trimming as a prevention of lameness*.”

A number of farmers felt that knowledge and training of prevention methods was an issue:

“*Lack of knowledge of prevention techniques*”.

And

“*Knowing what to do and when*.”

A number of farmers simply stated that lameness was not “a big problem” on their farm, with one farmer stating:

“*If it isn't broke, don't try to mend it*”.

And another, when discussing the issues with prevention pointed out that they were:

“*Not looking for any more work or expense*.”

However, one farmer stated that the only reason for not preventing lameness was:

“*Just laziness of owners*.”

Lastly, with respect to theme C, over a quarter of respondents stated that nothing stops them from preventing lameness, or makes prevention difficult, with one farmer clarifying:

“*Prevention of lameness is a priority, and I would seek advice when needed*.”

### Farmer Training

In order to investigate farmers' training, participants were presented with five lameness-related topics: (i) How to trim feet, (ii) Locomotion/mobility scoring, (iii) How to prevent lameness, (iv) Recognition of different foot conditions, and (v) How to treat lameness. The farmers were asked about their source of training (if any) and requested to select the one answer that best applied to their situation for each of these five topics. The possible responses for this question were (a) “I have received specific training (e.g., from a foot trimmer or at college)”, (b) “I am self-trained”, or (c) “I have had no training'. A few farmers selected two answers for some topics and these responses were excluded from analysis (*n* = 24 responses from eight individual farmers).

**Figure 10:**
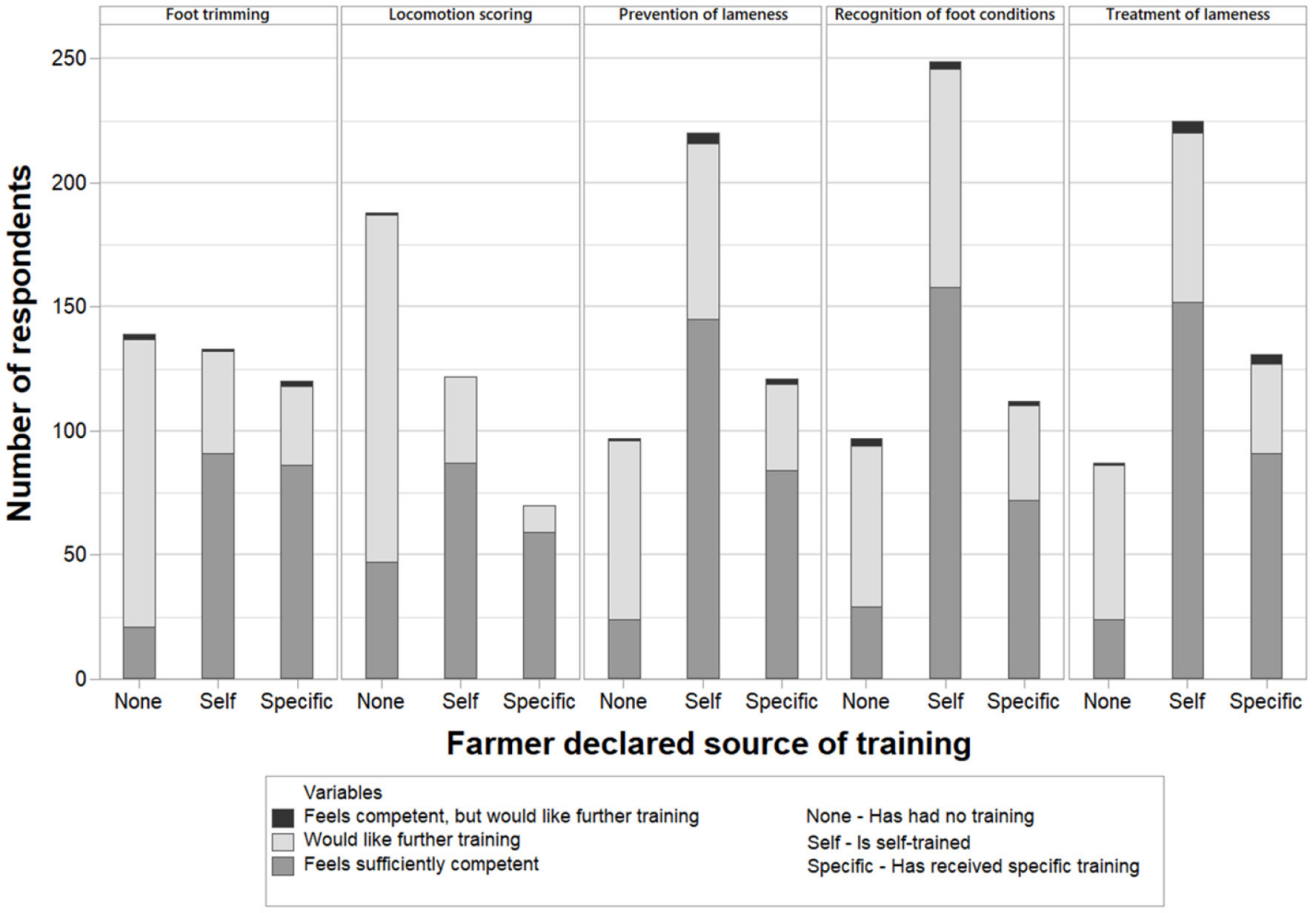
Farmer self-reported source of training regarding five lameness topics, with corresponding declaration of either feeling sufficiently competent, wanting further training, or feeling competent but wanting more training. The five topics - shown from left to right - were: Foot trimming (*n* = 392), Locomotion/mobility scoring (*n* = 380), Prevention of lameness (*n* = 438), Recognition of different foot conditions (*n* = 458) and Treatment of lameness (*n* = 443). As farmers could choose which questions to answer, the number of respondents differed between topics.

On the same five lameness-related topics, the farmers were then asked to select from the two options (a) “I feel sufficiently competent” and (b) “I would like further training,” and could select both if they wished. Figure 10 displays how responses on previous training related to views on competency and training desire (for those respondents that answered both questions on a topic).

### Associations Between Responses

Female respondents were less likely to declare that they felt sufficiently competent at foot trimming (Fisher's exact *p* < 0.001) or lameness prevention (Fisher's exact *p* = 0.016). Respondents who reported that their main source of income is not derived directly from livestock or agriculture were more likely to declare that they had <10 suckler cows (Fisher's exact *p* = 0.002), and <10 weaned animals being reared for beef (Fisher's exact *p* < 0.001) than respondents declaring one of several options where agriculture was the main source of income. They were also less likely to select that it was possible, and safe, to pick up front (Fisher's exact *p* < 0.001) or back feet (Fisher's exact *p* < 0.001) and were also less likely to always treat lame animals themselves (Fisher's exact *p* < 0.001). In addition, they were more likely to have had no training in recognition of different foot conditions (Fisher's exact *p* < 0.001), foot trimming (Fisher's exact *p* < 0.001), lameness treatment (Fisher's exact *p* < 0.001), or lameness prevention (Fisher's exact *p* < 0.001). Again, using Fisher's exact test, the same respondents were more likely to state that they would like further training in the recognition of different foot conditions (*p* = 0.001), foot trimming (*p* < 0.001), lameness treatment (*p* < 0.001), lameness prevention (*p* < 0.001), and locomotion scoring (*p* = 0.001).

## Discussion

### Lameness Prevalence

Farmers reported a mean farm level lameness prevalence of 2% for suckler herds and 0.6% for finishing units. This contrasts with earlier unpublished work by the authors where a mean farm level lameness prevalence of 14.2% (range 0 to 43.2%) for suckler farms and 8.3% (range 2.0 to 21.2%) for finishing units was identified in the UK by JT locomotion scoring. However, there is evidence in both beef and dairy settings that farmers tend to estimate a lower prevalence of lameness than researchers (20, 21). This could, in part, be due to the lack of locomotion scoring or due to the method of lameness detection in general. That there may be a level of discrepancy on classifying and defining a lame animal between researchers and farmers, including what terms or language are used, has been confirmed in the authors' earlier interview study of beef farmers (21). In these regards, research suggests that some farmers have difficulties detecting lame animals because of poor facilities, especially problems relating to finding a suitable place to conduct the locomotion scoring (21), or due to a lack of training (26). Recall bias may have affected the numbers provided in the responses, especially if lame animals are not recorded. No matter what the reason for the lower estimates, if it is the case that lame animals are not being identified, this will be a barrier to treatment of individual animals. This would also mean that the full extent of the lameness problem was not acknowledged or recognized by the farmer and this would have implications for taking preventative measures at a herd level.

### Lameness Identification, Examination, and Treatment

Over 85% of responding farmers reported that picking up the feet of lame animals was either not safe or not possible. This is concerning for three main reasons: first, because many types of lameness require the foot to be lifted in order to make a diagnosis and treat the condition; second, because of the perceived difficulties previously identified in getting professional foot trimmers to examine individual lame animals promptly (21), the importance of farmers being able to safely pick up the feet of animals themselves becomes more apparent; and third, because prompt treatment is very important with respect to both prognosis and protecting animal welfare (27).

The difficulty in lifting feet may in part explain the low number of farmers that use foot blocks, or bandages, as these require suitable facilities (specifically a suitable crush and race). The motivators of the 19% of respondents stating that they always give antibiotics when treating lame animals may be an area for further investigation. Especially considering that some of these may not be lifting feet, and so a diagnosis supporting antibiotic use as appropriate may not have been reached. Earlier unpublished work by the authors suggest that claw horn lesions are more prevalent in UK beef cattle, for which antibiotics will offer no benefit (unless complicated by secondary infections).

NSAIDs were used at least sometimes by 85% of farmers, which is likely to be important for alleviating pain, and for recovery. Previous studies have shown that the administration of NSAID augments other treatments for claw horn disease (25) and appears to reduce post-treatment hypersensitivity (28).

Antibiotic choice suggests that the most commonly used products are from European Medicines Agency (EMA) category D, which is the category to be used prudently as a first-line treatment (29). Although the UK has not fully adopted this categorization, it provides a useful basis to evaluate the farmer-reported use of antibiotics. There were a number of products in category C, namely, macrolides, amoxicillin and clavulanic acid, dihydrostreptomycin, and florfenicol, which, under the guidance, should be used with caution, although some of which are licensed for use to treat lame cattle. Very few farmers used a category B drug (ceftiofur, stated by four respondents), which, under the guidance, should be restricted, although there are ceftiofur products licensed for treating causes of lameness in cattle. Injectable macrolides (stated as being used by 15% of farmers) may be attractive because of the long-acting nature of some products in this group. A potential concern is that three farmers suggested they used tilmicosin, despite this product being restricted to veterinary administration only. Some of these respondents may be both farmers and veterinary surgeons, or they may have misunderstood the question. While the common selection of category D drugs is reassuring in the context of critically important antimicrobials, the findings on antibiotic usage per se show an opportunity for improved veterinary input on appropriate drug use.

Foot baths were used as a treatment by just under a third of respondents, and the popularity of products used contrasted with a dairy farming study in the United States, where copper sulfate was the predominant product, with formaldehyde used by just 7.7% (30). Antibiotics were used by 17% of farmers in the same study, compared to 5% of respondents in this study. Solely using a water foot bath was suggested by a small number of farmers in this study. This may be intended to clean the hoof. However, due to the lack of any disinfectant capability, there is a risk that this may spread infection. Over 65% of farmers reported that they do not use a foot bath. This may be because of a difficulty in providing a foot bath to beef cattle, both when at grass and when housed (21), with less occasions of routine journeys through farm buildings or handling systems in beef units compared to dairy herds. There is limited literature regarding optimal foot bath products, but antibiotics and disinfectants such as formaldehyde and copper sulfate are generally considered beneficial for the control of lameness (31–33). However, the use of antibiotics in foot baths constitutes off-license application in the UK, and under the responsible use of antimicrobials aspect, their use is difficult to justify (34). While the number of farms using antibiotic foot baths was comparatively small in our study, their use as such for this purpose again highlights that there is room for improved veterinary input on drug use. Formaldehyde is classed as a potential carcinogen, and copper sulfate is not degraded in the environment, and as such the future availability of each of these products is uncertain.

Of particular concern were the 4% of respondents who supported the statement “I never treat lame animals, as they get better by themselves,” as well as the 16% of respondents who selected the option to “wait a week or two before examining lame animals to see how they do,” and the 35% of farmers that expressed that they only examine lame animals if they are “walking quite badly.” These approaches to a lameness case are a concern for two main reasons. Firstly, they are likely to leave animals in pain, and secondly, this may lead to more severe lesions and affect the recovery potential (27). We speculate that perhaps one reason for taking these types of approaches to dealing with lameness stems from a lack of knowledge by farmers on the consequences of such actions. However, the 59% of farmers who agreed, or strongly agreed, that they “pick up the foot of a lame animal within 48 h” should be considered a positive sign. This wide variation between farmers' approaches compares with the earlier interview findings of UK beef farmers (21).

When farmers were asked to select options for dealing with ongoing lameness cases, no information about severity of lameness or chronicity was collected. However, the 35% of respondents that selected that they can transport the animal to slaughter remains an approach that divides opinion (2, 35). Farmers must bear in mind UK regulation requiring that animals must not be transported “in a way likely to cause them injury or undue suffering” (36). The animal must also bear weight on all four limbs when standing or walking and stand up unaided under fitness to travel rules (37). These requirements are likely to preclude many cases of lameness from being transported, although there are very limited exceptions for “slightly injured or ill” animals with Official Veterinarian agreement, provided improved transport conditions and direct sending for immediate slaughter are met (38).

Only 40% of farmers in this study thought that on-farm euthanasia and disposal by the knackerman/hunt kennel was an option available to them. However, for animals with chronic lameness who are in pain and not responding to treatment, this is an option for all farmers (because in the UK, on-farm euthanasia and disposal as a service is available for all geographical areas), and in some cases, this may be the only option to preserve welfare. Over a third of responding farmers selected that they might call the veterinarian to request an emergency slaughter certificate. For lameness cases, it is likely that the veterinarian will be unable to provide a certificate, because the condition of “a healthy animal that has suffered an accident” is not met (38). Stojkov et al. (39) conducted a study at Canadian dairy cull cow markets and identified that almost a third of cows at these livestock markets had poor fitness for transport. It is unknown to what extent this problem may occur in the UK.

Farmer-reported barriers to both treatment and prevention of lameness were largely similar. The questions did follow sequentially, so some order effect may have occurred. However, the results are similar to findings in the authors' earlier interview study of beef farmers (21), where poor handling facilities (especially not being able to foot bath animals, or lift their feet safely or at all), shortage of staff (especially when tasks required two people, but only one was available), lack of time, and suboptimal knowledge of farmers regarding lameness control and prevention were all important concerns for farmers. This suggests that these are important areas for improvement, with routes to achieve this potentially including raising awareness, better understanding incentives and motivations, and offering alternative sources of support and knowledge exchange.

### Farmer Training

Farmers reported a large proportion of self-training in lameness-related topics. Despite this, over half of this group self-reported to be sufficiently competent in each topic. There was also a large proportion of farmers who reported to have no training, yet still felt sufficiently competent. This may be because they do not feel that they need to be trained in it, perhaps relying on expert advice or getting professionals to perform lameness-related tasks instead, or because they perceive it to be unimportant. An animal welfare concern would be unconscious incompetence, where they feel competent, but are not, and this may apply to some respondents.

However, a high proportion of farmers did select that they would like further training in each of the five topics. This ties in with the farmers' own self-reported barriers to the treatment and prevention of lameness (described in the previous section) relating to their lack of knowledge and training. Some farmers selected that they felt sufficiently competent, but still wanted further training. This may be due to either a general desire for knowledge, or a belief that, although they deem their current ability as satisfactory, further training may improve their skills. There may also be an element of social desirability bias occurring, such that some may be reluctant to admit that they are not sufficiently competent given that they are treating animals themselves, and therefore self-report that they are competent. Social desirability bias can occur regardless of the fact that the questionnaires where returned anonymously. These findings compare to earlier interview findings (21), where some farmers reported that they did not feel confident enough to trim feet, and others displayed little knowledge of lesion recognition. Further training to those who would like it could lead to faster recognition and treatment of lameness and improved welfare and production, and this study supports the notion that there is demand by beef farmers for more training in lameness prevention and control.

Of particular interest is the high likelihood of respondents with a small herd (<10 breeding cows or weaned cattle reared for beef) to declare having had no training and desiring further training, compared to those with larger herds. These farmers with small herds may make prompt, appropriate use of professional services. However, with evidence of some farmers having difficulties accessing foot trimmers when only presenting a small number of cattle (21), and these farmers being less likely to be able to safely lift the feet of lame animal themselves, there is a real risk of lame animals in such small herds being left untreated or incorrectly treated.

### Representativeness of Responses and Respondents

Data from the UK Cattle Yearbook 2019 (using 2017 data) indicate that the number of non-dairy holdings across the UK is 61,460, with a distribution of 45% in England, 12% in Wales, 16% in Scotland, and 27% in Northern Ireland (40). If it was assumed that all these holdings were eligible for participation, ~0.9% of farmers were sampled. These questionnaire responses are biased toward farmers in England, with 87% of respondents in England. Wales was almost proportionately represented with 11% of respondents, but Scotland and Northern Ireland were underrepresented (2%). This is not unexpected, with addresses for beef farmers in Scotland and Northern Ireland not available for a directed distribution, but this means that our sample is not representative of farms in these regions. The year book data suggest that the mean beef cow herd sizes are 27 for England, 48 for Scotland and 18 for Northern Ireland (no data for Wales) (40), which is comparable to the mean number of cows on farms of respondents, which was 50. The spread of respondents across English regions was considered acceptable, having responses from all regions but London. Age demographics for UK livestock keepers are not available; however, the median age of registered agricultural holders (the person in whose name a holding is operated) in the UK is 60 years, with those 55–64 years of age representing 36% of holdings in 2016 (41). This compares to 29% of respondents to this questionnaire being in the 56–65 years of age category.

The UK beef industry utilizes diverse production systems, with considerable variation in breeds (purebred and crossbred animals, including dairy-origin animals), in target finishing ages (common range of 12–30 months old), in grazing and housing management (permanent pastures and temporary leys; zero-grazing, all-year grazing, to a combination of winter housing and summer grazing), in nutrition (forage and cereal-based rations), and in the main purpose of the farm (including breeding to weaning, breeding to finishing, rearing store cattle, or only finishing cattle) (2, 38). This study did not endeavor to capture the precise setup of each respondent, because the variation and lack of comparison base would have added little robust value to the analysis.

### Limitations

When interpreting the results of this study, it must be recognized that the nature of a voluntary questionnaire may lead to a non-response bias, as those choosing to respond may have differed in some way to those who chose not to respond. We could not accurately estimate the response rate for this survey. Selection bias may have also occurred, as online circulation will have favored those farmers with access to, and more regular use of online media. In addition, the first launch of the paper questionnaire was sent to all AFU addresses in England and Wales, and so was biased toward farmers with finishing units, who had reason to register their holding as an AFU. The list of farm addresses used for the stratified sample to receive a postal questionnaire only included holdings in England and Wales, which added selection bias. There was also a risk of recall bias in naming products used and stating the number of lame animals believed to be on farm. All questions were asked in the same order, although for online respondents, row items in scale questions and options in multiple choice questions were randomized unless the options had an inherent order. This may have introduced an order bias, for example, by asking farmers about their handling facilities before asking about difficulties in treating lame animals. In addition, there may have been an element of social desirability bias, whereby respondents may have wanted to provide a perceived “correct” answer. Farmers could choose not to answer questions and this has been made clear in the results selection, with the number of respondents for each question given in brackets. We highlight that less farmers answered the questions on different treatments for lameness and we do not know the reason for this. It was possible for more than one person to answer the questionnaire per farm, but we do not know if this occurred or to what extent. If it occurred, our data would contain some clustering and the statistical tests for associations would be less robust. Despite these limitations, and the representative issues pertaining to our sample (as discussed in the previous section), this questionnaire is still a useful tool to capture findings for a large number of farmers, and, in the authors' opinion, these results provide important information regarding farmer perceptions and protocols.

## Conclusions

This research identified UK beef farmers' perceived lameness prevalence on their farms to be generally low, with previous work suggesting that this may be an underestimation. Approaches to dealing with lameness prevention and control are extremely variable among UK beef farmers, and farmers acknowledged a need for further training relating to lameness. Important themes posing barriers to lameness treatment and prevention were (i) facilities and location of cattle, with over 50% of UK beef farmers in this study unable to lift all four feet safely, and (ii) shortage of skilled staff, lack of time, and suboptimal knowledge base. As an example for the second theme, over 90% of respondents did not locomotion score, and so may not identify a problem, where one exists. This potential lack of identification may explain the possible underestimation of lameness prevalence that may have been seen here and is a critical barrier to a farmer instigating both treatment and prevention plans. Additionally, farmer awareness of appropriate options to deal with ongoing lameness cases is a concern. For example, do the 35% of participants that consider transporting lame animals to slaughter as an option understand the regulations and requirements for transport, and is this option applied to inappropriate cases? Future work to identify how best to support farmers, with knowledge exchange regarding approaches to treating and preventing lameness, as well as training in these areas has the potential to improve both animal welfare and farm productivity.

## Data Availability Statement

The raw data supporting the conclusions of this article will be made available by the authors, without undue reservation.

## Ethics Statement

The studies involving human participants were reviewed and approved by University of Liverpool Research Ethics Committee (VREC 533). Written informed consent from the participants' legal guardian/next of kin was not required to participate in this study in accordance with the national legislation and the institutional requirements.

## Author Contributions

JT designed the interview script, piloted the script, recruited the participants, analyzed the data, and wrote the manuscript. HMH conceived the original study and assisted in designing the interview script, analysis of the data, and critical appraisal of the manuscript. KM conceived the original study, is PI on the grant, assisted in designing the interview script and analysis of the data, and critically appraised the manuscript. DG-W and JO appraised both the original study design and the final manuscript. All authors contributed to the article and approved the submitted version.

## Conflict of Interest

The authors declare that the research was conducted in the absence of any commercial or financial relationships that could be construed as a potential conflict of interest.
