# Supplementary material for: Lameness in Beef Cattle: A Cross-Sectional Descriptive Survey of On-Farm Practices and Approaches
Source: Front Vet Sci. 2021 Jun 4;8:657299. doi: 10.3389/fvets.2021.657299 (PMC8212986; doi:10.3389/fvets.2021.657299)
Supplement: Supplementary file 1 [file Data_Sheet_1.docx]

Appendix 1 – Supplementary material: Printed Questionnaire

Lameness in Beef Cattle


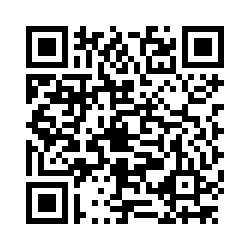

My name is Jay Tunstall, and I am a farm vet undertaking research at The University of Liverpool about **lameness in beef cattle**. Please read on if you are involved with beef cattle farming: **breeding heifers or cows**, or weaned cattle that are being reared for **beef** production (including**stores, fattening, finishing**). Please continue, no matter whether you feel lameness affects the cattle you're involved with or not.
 
Your responses will be **anonymous**, and you do not need to leave your name or other details if you do not wish to do so. You will have the option to enter a **prize draw**.  If you enter the draw, your details will be used solely for the purposes of the prize draw.
 
For most questions, we will ask you simply to **tick boxes**. There are opportunities to leave additional comments, and we very much welcome these. The questionnaire should only take about 10 minutes to complete.
 
If you would prefer to complete an online version of the questionnaire, please use the link below, or scan the QR code below.

www.bit.ly/beef-lameness

Many thanks for your help with this study.

 ​For more details on the study, please see Participant Information Sheet.
 ​

**Please select the option below to confirm that you have read the Participant Information Sheet and consent to participation in the study, and also that you understand:**

•That you will not be identifiable from any data used in this study
•That you may withdraw at any time without giving a reason and without incurring a disadvantage
•That you may request destruction of the data you supply, up until the point at which it has been anonymised
•That you agree to the use of the information provided for this study and for future research

- **I have read, understand and agree**
- **I have not read, understood, or I do not agree**

**If you are involved with more than one beef unit (breeding, rearing or finishing), please answer all questions for the unit that you have the most 'hands on' involvement with the cattle.**
If you are **not** involved with beef cattle, thank you for your interest, but this questionnaire is not applicable to you.

**Q1** When thinking about lifting the **FRONT** feet of beef cattle, how would you best describe the facilities on farm?
Please select one answer, and don't include facilities brought in by a contractor / foot trimmer / vet.​

- Lifting and examination is possible, but is generally NOT safe for either the animal or the person
- Lifting and examination IS possible and generally safe for animal and person
- Lifting and examination is NOT possible

**Q2** When thinking about lifting the **BACK** feet of beef cattle, how would you best describe the facilities on farm?
 Please select one answer, and don't include facilities brought in by a contractor / foot trimmer / vet.​

- Lifting and examination is possible, but is generally NOT safe for either the animal or the person
- Lifting and examination IS possible and generally safe for animal and person
- Lifting and examination is NOT possible

**Q3** Please add any additional comments in the space below.

________________________________________________________________

________________________________________________________________

________________________________________________________________

________________________________________________________________

**Q4** Do you treat lame beef cattle yourself?

- Always
- Sometimes
- Never (if never, please skip to **Q10**)

**Q5** When **you** treat lame beef cattle, what treatments do **you** use?
Please select one answer per row.

|  | Never | Sometimes | Often | Always | Unsure |
| --- | --- | --- | --- | --- | --- |
| Antibiotic product, applied onto foot |  |  |  |  |  |
| Antibiotic product given by injection |  |  |  |  |  |
| Pain relief / anti-inflammatory product (Veterinary product, e.g. Metacam / Ketofen) |  |  |  |  |  |

**Q6** When **you** treat lame beef cattle, what other treatments do **you** use?
 Please select one answer per row.

|  | Never | Sometimes | Often | Always | Unsure |
| --- | --- | --- | --- | --- | --- |
| Foot block |  |  |  |  |  |
| Foot bath |  |  |  |  |  |
| Bandage / wrap |  |  |  |  |  |

**Q7** Please add **any other treatments** or additional comments in the space below.

________________________________________________________________

________________________________________________________________

**Q8** Please tell us the two most common antibiotic injection products you use when you treat lame beef cattle.

________________________________________________________________

________________________________________________________________

**Q9** Please tell us what products you use in a foot bath.

________________________________________________________________

________________________________________________________________

**Q10** What options do you feel are available to beef farmers to deal with animals that have ongoing lameness?
Please select as many answers as you feel are available to beef farmers.

- Call knackerman or hunt kennel for collection and disposal
- Arrange treatment and keep the animal on farm
- Transport to slaughterhouse
- Call the vet for emergency slaughter certificate / on farm slaughter
- Monitor animal and allow time to recover, without treatment
- None of the above

**Q11** Please add any other options you feel are available to beef farmers to deal with animals that have ongoing lameness.

________________________________________________________________

________________________________________________________________

________________________________________________________________

________________________________________________________________

**Q12** Have you had any training on lameness?
Please select the one option that best applies to your situation for each of the topics below.

|  | I have had no training | I am self-trained | I have received specific training (e.g. foot trimmer, college) |
| --- | --- | --- | --- |
| Recognition of different foot conditions |  |  |  |
| How to trim feet |  |  |  |
| How to treat lameness |  |  |  |
| How to prevent lameness |  |  |  |
| Locomotion / mobility scoring |  |  |  |

**Q13** Please write any comments in the space below.

________________________________________________________________

________________________________________________________________

**Q14** For each topic below, please select all options that apply
Please select options that apply.

|  | I feel sufficiently competent | I would like further training |
| --- | --- | --- |
| Recognition of different foot conditions |  |  |
| How to trim feet |  |  |
| How to treat lameness |  |  |
| How to prevent lameness |  |  |
| Locomotion / mobility scoring |  |  |

**Q15** Please write any comments in the space below.

________________________________________________________________

________________________________________________________________

________________________________________________________________

________________________________________________________________

**Q16** How many beef cattle of each type are on the farm, and how many of each would you say are currently lame?
Please complete all four boxes (using 0 if none).

|  | How many animals? | How many lame? |
| --- | --- | --- |
| Breeding (suckler) cows including in-calf heifers |  |  |
| Animals being reared for meat, from weaning up to slaughter |  |  |

**Q17** Please add any comments in the space below.

________________________________________________________________

________________________________________________________________

________________________________________________________________

________________________________________________________________

**Q18** Following discussions with beef farmers, the following are all approaches that might be taken to deal with lame animals.
Please select one answer per row to indicate your level of agreement or disagreement.​

|  | Strongly agree | Agree | Neither agree nor disagree | Disagree | Strongly disagree |
| --- | --- | --- | --- | --- | --- |
| I pick up the foot of a lame animal within 48 hours |  |  |  |  |  |
| I never deal with lame animals, as they get better by themselves |  |  |  |  |  |
| I give lame animals a week or two before examining them, to see how they do |  |  |  |  |  |
| I personally never pick up feet, but I get my vet or foot trimmer to do it as soon as possible |  |  |  |  |  |
| I only examine animals if they are walking quite badly |  |  |  |  |  |
| I ask the vet to look at a lame animal, but only if the vet happens to be on farm already |  |  |  |  |  |

**Q19** Please add any comments in the space below.

________________________________________________________________

________________________________________________________________

________________________________________________________________

________________________________________________________________

**Q20** Do you mobility score / locomotion score beef cattle on the farm?

- No (If No, please skip to **Q22**)
- Yes
- Unsure

**Q21** How **often** do you mobility score / locomotion score and **how** exactly do you usually do it?

________________________________________________________________

________________________________________________________________

________________________________________________________________

________________________________________________________________

**Q22** What, if anything, prevents you treating lame beef cattle, or makes treatment difficult?

________________________________________________________________

________________________________________________________________

________________________________________________________________

________________________________________________________________

**Q23** What, if anything, stops you preventing lameness in beef cattle, or makes prevention difficult?

________________________________________________________________

________________________________________________________________

________________________________________________________________

________________________________________________________________

**Thank you for helping us to understand lameness on your farm. The survey is almost complete. Please could you now answer a small number of questions to help us understand you and your farm.**

**Q24** When considering the beef cattle part of your farm, would you consider yourself responsible for:
Please select all answers that apply.

- Long term farm planning
- Day to day management decision making
- Day to day stockmanship / animal care

**Q25** Which of the following best describes you?
Please select one answer.

- Beef farming is my main source of income
- Arable farming is my main source of income
- Beef farming provides an equal top share of my income with another source
- Livestock, but NOT beef farming, is my main source of income
- My main source of income is NOT derived directly from livestock or agriculture

**Q26** How would you describe yourself?
Please select all answers that apply.

- Farm owner
- Farm manager
- Farm worker
- Other (please specify) ________________________________________________

**Q27** Please select your age

- 15 or less
- 16 - 25
- 26 - 35
- 36 - 45
- 46 - 55
- 56 - 65
- 66 - 75
- 76 or over
- Prefer not to say

**Q28** Please select your sex

- Male
- Female
- Other
- Prefer not to say

**Q29** Please state the county that your farm is in (for example Herefordshire).
If your farm crosses boundaries, please consider the county within which most cattle are currently in.
This will not be used to identify you, but you may skip this question if you wish.

________________________________________________________________

________________________________________________________________

**Q30** Is rearing and selling beef **breeding** stock a major part of your business?

- No
- Yes
- Unsure

**Q31** Are your cattle classified as organic?

- No
- Yes
- Unsure

**Q32** Do you have any final comments on lameness, your involvement, or the farm?

________________________________________________________________

________________________________________________________________

________________________________________________________________

________________________________________________________________

Thank you for reaching the end of the survey. If you would like to be entered into the prize draw to win a pair of Muck Boot Company wellies, please leave your name, contact details and wellie size below.
Please note, these details will only be used in relation to the prize draw.

________________________________________________________________

________________________________________________________________

________________________________________________________________

________________________________________________________________

If you wish to receive a summary of the results of this questionnaire, please leave your name and postal or email address.

- Please use details provided for the draw
- Name and address _____________________________________________

________________________________________________________________

________________________________________________________________

________________________________________________________________

________________________________________________________________
